# Efficiency of different protocols for enamel clean-up after bracket debonding: an *in vitro* study

**DOI:** 10.1590/2177-6709.20.5.078-085.oar

**Published:** 2015

**Authors:** Lara Carvalho Freitas Sigilião, Mariana Marquezan, Carlos Nelson Elias, Antônio Carlos Ruellas, Eduardo Franzotti Sant'Anna

**Affiliations:** 1 Dentist, Brazilian Navy, Rio de Janeiro, Rio de Janeiro, Brazil; 2 Postdoc resident in Orthodontics, Universidade Federal do Rio de Janeiro (UFRJ), Rio de Janeiro, Rio de Janeiro, Brazil; 3 Professor, Instituto Militar de Engenharia (IME), Rio de Janeiro, Rio de Janeiro, Brazil; 4 Professor, Universidade Federal do Rio de Janeiro (UFRJ), Rio de Janeiro, Rio de Janeiro, Brazil

**Keywords:** Orthodontic brackets, Dental enamel, Dental debonding

## Abstract

**Objective:**

This study aimed to assess the efficiency of six protocols for cleaning-up tooth enamel after bracket debonding.

**Methods:**

A total of 60 premolars were divided into six groups, according to the tools used for clean-up: 12-blade bur at low speed (G12L), 12-blade bur at high speed (G12H), 30-blade bur at low speed (G30L), DU10CO ORTHO polisher (GDU), Renew System (GR) and Diagloss polisher (GD). Mean roughness (Ra) and mean roughness depth (Rz) of enamel surface were analyzed with a profilometer. Paired t-test was used to assess Ra and Rz before and after enamel clean-up. ANOVA/Tukey tests were used for intergroup comparison. The duration of removal procedures was recorded. The association between time and variation in enamel roughness (∆Ra, ∆Rz) were evaluated by Pearson's correlation test. Enamel topography was assessed by scanning electron microscopy (SEM).

**Results:**

In Groups G12L and G12H, original enamel roughness did not change significantly. In Groups G30L, GDU, GR and GD, a smoother surface (*p* < 0.05) was found after clean-up. In Groups G30L and GD, the protocols used were more time-consuming than those used in the other groups. Negative and moderate correlation was observed between time and (∆Ra, ∆Rz); Ra and (∆Ra, ∆Rz); Rz (r = - 0.445, r = - 0.475, *p* < 0.01).

**Conclusion:**

All enamel clean-up protocols were efficient because they did not result in increased surface roughness. The longer the time spent performing the protocol, the lower the surface roughness.

## Abstract (pt)

**Objetivo:**

esse estudo objetivou avaliar a eficiência de seis protocolos de remoção de resina do esmalte após a descolagem de braquetes.

**Métodos:**

sessenta (60) pré-molares foram divididos em seis grupos conforme as ferramentas utilizadas: broca de 12 lâminas em baixa rotação (G12L), broca de 12 lâminas em alta rotação (G12H), broca de 30 lâminas em baixa rotação (G30L), polidor DU10CO-ORTHO (GDU), Renew System (GR) e polidor Diagloss (GD). As médias de rugosidade (Ra) e profundidade média de rugosidade (Rz) da superfície do esmalte foram analisadas com perfilômetro. Teste *t* pareado foi utilizado para avaliar Ra e Rz antes e depois da limpeza do esmalte; testes de ANOVA/Tukey foram utilizados para avaliar a diferença intergrupos. A duração dos procedimentos de remoção da resina foi registrada. Ainda, a associação entre o tempo e a variação da rugosidade do esmalte (ΔRa, ΔRz) foi avaliada pelo teste de correlação de Pearson. A topografia do esmalte também foi avaliada, por microscopia eletrônica de varredura (MEV).

**Resultados:**

nos grupos G12L e G12H, a rugosidade do esmalte original não se alterou significativamente. Nos Grupos G30L, GDU, GR e GD, foi verificada superfície mais lisa após a limpeza (*p *< 0,05). Nos Grupos G30L e GD, os protocolos utilizados foram mais demorados que nos demais grupos. Foi observada correlação negativa e moderada entre tempo, ΔRa e ΔRz (r = -0.445, r = -0.475, *p*< 0,01).

**Conclusão:**

todos os protocolos de limpeza do esmalte foram eficientes, pois não resultaram no aumento da rugosidade superficial. Quanto maior o tempo gasto, menor a rugosidade da superfície.

## INTRODUCTION

Direct bracket bonding to tooth surface became possible with the advent of acid etching which revolutionized the orthodontic practice.^1^ On completion of orthodontic treatment, the residual resin left behind after bracket debonding must be cleaned efficiently and rapidly while preserving enamel surface; in addition, enamel surface must be smoothed and polished to prevent plaque accumulation. Several factors are involved in these procedures, including the tools used for debonding, protocols for residual resin removal, the type of adhesive used^2^ and the operator's skill.

Although there is no consensus in the literature regarding this matter, one of the most common methods of removing residual adhesive from the enamel surface is using a tungsten carbide bur at low speed.^3^^-^ 6 Several new and more conservative multiple and one-step systems for enamel clean-up, such as fiber-reinforced composites,^7^ polishers with diamond particles, aluminum oxide rubber and sandblasting,^6^ have been developed and gained popularity among orthodontists. However, many of these tools have not been tested as a method of providing characteristics similar to those of the original enamel. 

The aims of this study were to compare *in vitro* enamel surface roughness by using six protocols for removal of adhesive remnant and enamel polishing after bracket debonding; assess the time spent to remove residual resin in each one of them; and assess the correlation between roughness and removal time.

## MATERIAL AND METHODS

This study was approved by the Research and Ethics Committee of the Institute of Public Health and Research at Universidade Federal do Rio de Janeiro, Brazil (#05/2012).

A total of 60 human caries-free premolars extracted for orthodontic purposes were stored in aqueous solution of thymol (0.1%) to prevent bacterial growth and dehydration. Teeth were selected based on visual observation of soundness of the buccal surfaces, absence of caries and cracks in the coronal portion, and no previous exposure to adhesive agents. The teeth roots were removed and the crowns were embedded in self-polymerizing acrylic resin with the buccal surfaces facing upwards. The bond area was limited by marks made on the base of the specimens to ensure that roughness assessments were made in the same area.

Samples were randomly divided into six equal groups (n = 10) to compare different protocols for removal of adhesive remnant and enamel polishing (Table 1). Sample size was calculated at a level of significance set at 5% and test power of 80%, based on data from a previous study.^8^

**Table 1:** - Distribution of groups according to the protocol applied for removal of adhesive remnant.

**Groups**	**N**	**Protocols**
G12L	10	12-blade tungsten carbide bur (low speed)^a^
G12H	10	12-blade tungsten carbide bur (high speed)^b^
G30L	10	30-blade tungsten carbide bur (low speed)^c^
GDU	10	DU10CA ORTHO Points^d^
GR	10	12-blade tungsten carbide bur (high speed) + Renew(tm) Finishing System Point^e^
GD	10	Diagloss polisher^f^

^a^ Ref. H23R.21.012 (Brasseler^(r)^, Savannah, GA, USA), 20,000 rpm;

^b^ Ref. H23R.31.012 (Brasseler^(r)^, Savannah, GA, USA);

^c^ Ref. FF9714 ( Jet - Beavers Dental^(r)^, Ontario, Canada), 20,000 rpm;

^d^ DU10CA ORTHO (DhPro^(r)^, Paranaguá, PR, Brazil), 9,000 rpm;

^e^ Renew(tm) Finishing System (Reliance Orthodontics^(r)^ - Illinois, USA);

^f^ Diagloss polisher (Edenta, Switzerland), 10,000 a 12,000 rpm

Teeth were cleaned with fine pumice slurry using a rubber cup in a low-speed handpiece for approximately 10 seconds, followed by rinsing and drying with moisture-free air spray. Subsequently, teeth were etched for 20 seconds with 37% phosphoric acid gel (Magic Acid Vigodent^(r)^, Rio de Janeiro, RJ, Brazil), rinsed for 20 seconds and dried. Premolar metal brackets (Morelli^(r)^, Sorocaba, SP, Brazil) were bonded to teeth with Transbond XT (3M Unitek, Monrovia, Calif, USA), following the manufacturer's instructions. Brackets were placed on teeth surfaces and firmly pressed into position for the base to fit perfectly, providing uniform resin layer in all specimens. After removing excess resin from the edges of bracket bases with the aid of a dental probe, teeth were light-polymerized for 10 seconds on each side of the bracket by means of a conventional LED curing unit (Optilight Max - Gnatus^(r)^, Ribeirão Preto, SP, Brazil). Specimens were then stored in artificial saliva at 37 ^o^C for 24 hours to facilitate maximum polymerization and hydration of the material.

Brackets were then removed by gently squeezing their mesial and distal wings with How Reto pliers.^9 ^Enamel surfaces were evaluated under Olympus SZ40 stereomicroscope (Olympus, Japan) under 15X magnification.^10^ They were classified according to the Adhesive Remnant Index (ARI)^11^: score 0 = no adhesive on enamel, score 1 = less than 50% adhesive on enamel, score 2 = more than 50% adhesive on enamel, score 3 = all adhesive remaining on enamel. Teeth were included in the experiment only if the most of resin remained on enamel surface after debonding (score 2 or 3), in order to allow adequate evaluation of all finishing protocols. Fortunately, none of the samples were excluded. Groups G12L, G12H, G30L and GD had five specimens classified as ARI score 2 and five specimens classified as ARI score 3. Groups GDU and GR had four specimens classified as score 2 and six specimens classified as score 3.

The same operator performed debonding and adhesive removal without water cooling, and with a new bur or rubber used after treating every two teeth. The overall extent of resin removal was determined by visual inspection under the light of an operative lamp. The time required for completion of each resin removal protocol was recorded in seconds with a digital chronometer.

Quantitative and qualitative enamel evaluations were performed. For quantitative evaluation, roughness was measured at two time points: before bonding, to establish initial roughness; and after debonding and removal of adhesive remnants with finishing and polishing protocols, to establish final roughness. A profilometer (Mitutoyo Surftest SJ-400, Japan), with a cut-off value of 0.8 mm, was used to measure the roughness profile of each surface. Two measurements were performed on each specimen, parallel to one another, traversing the entire 4-mm bonding surface. The mean value of the two measurements of each specimen was recorded. This process involved recording two roughness parameters: 1) Mean roughness (Ra), in µm, determined as the arithmetic mean of all absolute distances of the roughness profile from the center line within the measuring length; and 2) Mean roughness depth (Rz) which describes the average maximum peak-to-valley height of five consecutive sampling lengths.^5^^,^ 12 Variation in roughness was calculated by the equations: ΔRa = final Ra - initial Ra and ΔRz = final Rz - initial Rz.

For qualitative evaluation of enamel surface, scanning electron microscopy (Quanta Feg 250, FEI Company, Oregon, USA) was performed to compare enamel surface of experimental groups.

## STATISTICAL ANALYSIS

Results were collected and statistically analyzed by means of SPSS version 20.0 software (Statistical Package for Social Sciences, SPSS Inc., Chicago, IL, USA). Distribution of variables was assessed for normality by Kolmogorov-Smirnov and Shapiro-Wilk tests. Paired t-test was used to assess the mean values ​​of roughness parameters (Ra and Rz) before and after enamel surface clean-up, and verify whether this processes altered enamel surface roughness. Intergroup differences for ΔRa, ΔRz and time required for cleaning the residual resin after bracket debonding were assessed by ANOVA/Tukey tests. Pearson's correlation test was performed to assess the association between ΔRa and ΔRz and time spent on each enamel clean-up protocol. A level of significance of 0.05 was used for all analyses.

## RESULTS

Results showed that all protocols tested for removal of adhesive remnant from enamel did not lead to increase in the original surface roughness significantly.

Ra results for measurements taken before bracket bonding and after residual resin removal are summarized in Table 2. Groups G12H and G12L, in which a 12-blade tungsten carbide bur was used at low and high speed, respectively, showed no significant differences before bonding and after debonding. Groups G30L, GDU, GR and GD showed a smoother surface after 30-blade tungsten carbide bur (low speed), DU10CA ORTHO points, 12-blade tungsten carbide bur (high speed) + Renew(tm) Finishing System, and Diagloss polisher were used, respectively (*p* < 0.05).

Rz results for measurements taken before bracket bonding and after residual resin removal are summarized in Table 3. Groups G12H and G12L showed no significant differences before bonding and after debonding, and so did Group GDU. Groups G30L, GR and GD showed a reduction in maximum peak-to-valley height (*p* < 0.05).

When ΔRa was compared by means of ANOVA/Tukey tests, there was no statistically significant difference among the six groups (Table 4). All values were negative because the final Ra value was lower than the initial Ra value. When the six groups were compared in terms of ΔRz, some statistical differences were observed (Table 4). Groups G30L and GD presented a decrease in vertical irregularities, while the positive value of ΔRz for G12H implied an increase in vertical irregularities.

**Figure 1:**
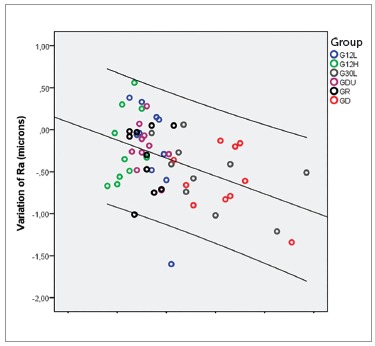
- Scatter plot of variation in roughness (DRa) in relation to time in all groups.

**Figure 2:**
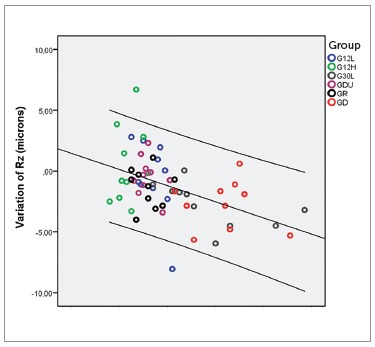
- Scatter plot of variation in roughness (DRz) in relation to time in all groups.

The time spent for resin remnant removal is shown in Table 5. The protocols used in Groups G30L and GD were more time-consuming than those used in the other groups (*p* < 0.05). Correlation between time-ΔRa and time-ΔRz was negative and moderate (Table 6). Scatter plots illustrate these results (Figs 1 and 2). 

**Table 2:** - Mean and standard deviation (SD) for initial and final Ra and results of paired t-test.

**Groups**	**Initial Ra (µm)**	**Final Ra (µm)**	***p*-value**
	**Mean (SD)**	**Mean (SD)**	
G12L	1.60 (0.50)	1.39 (0,15)	0.289
G12H	1.99 (0.34)	1.79 (0.38)	0.187
G30L	1.96 (0.50)	1.45 (0.43)	0.003 *
GDU	1.65 (0.34)	1.45 (0.24)	0.045 *
GR	1.64 (0.32)	1.31 (0.32)	0.025 *
GD	2.04 (0.43)	1.45 (0.22)	0.001 *

* Indicates statistical significance (*p* < 0.05).

**Table 3:** - Mean and standard deviation (SD) for initial and final Rz and results of paired t-test.

**Groups**	**Initial Rz (µm)**	**Final Rz (µm)**	***p*-value**
	**Mean (SD)**	**Mean (SD)**	
G12L	6.03 (3.04)	5.48 (0.59)	0.595
G12H	8.16 (2.16)	8.66 (1.75)	0.634
G30L	7.90 (2.33)	5.16 (1.77)	0.001*
GDU	6.26 (2.31)	5.82 (1.62)	0.404
GR	6.04 (1.50)	4.65 (1.00)	0.023*
GD	8.07 (2.47)	5.35 (1.06)	0.002*

* Indicates statistical significance (*p* < 0.05).

**Table 4:** - Mean and standard deviation (SD) for DRa and DRz and results of ANOVA/Tukey.

**Groups**	**DRa**	**DRz**
	**Mean (SD)**	**Mean (SD)**
G12L	- 0.20 (0.58)^a^	- 0.55 (3.15)^AB^
G12H	- 0.19 (0.43)^a^	0.49 (3.17)^B^
G30L	- 0.51 (0.39)^a^	- 2.74 (1.82)^A^
GDU	- 0.20 (0.27)^a^	- 0.44 (1.59)^AB^
GR	- 0.32 (0.38)^a^	- 1.39 (1.60)^AB^
GD	- 0.59 (0.38)^a^	- 2.71 (2.00)^A^

Each column indicates an independent statistical analysis. Different letters indicate statistically significant difference (*p* < 0.05) for ANOVA/Tukey.

**Table 5:** - Time required for cleaning residual resin after debracketing (seconds) *p* < 0.05.

	**G12L**	**G12H**	**G30L**	**GDU**	**GR**	**GD**
Mean (SD)	34.0 (5.73)	23.5 (5.01)	57.5 (19.9)	31.8 (4.56)	31.9 (5.85)	63.5 (13.8)
	A	A	B	A	A	B

SD - Standard deviation.

Different letters indicate statistically significant difference.

**Table 6:** - Pearson's Linear Correlation Coefficient between the time required and the variations in roughness.

	**DRa**	**DRz**
	(95% confidence interval)	(95% confidence interval)
Time	- 0.445 **	-0.475 **
	- (-0.685 _ -0.143)	(- 0.627 _ -0.214)

** *p* ≤ 0.01.

**Figure 3:**
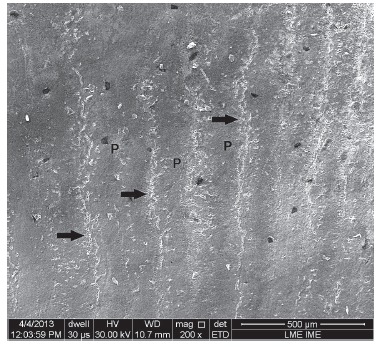
- Scanning electron microscopy (200 X magnification) of original enamel; perikymata (P); prism end openings (arrows).

**Figure 4:**
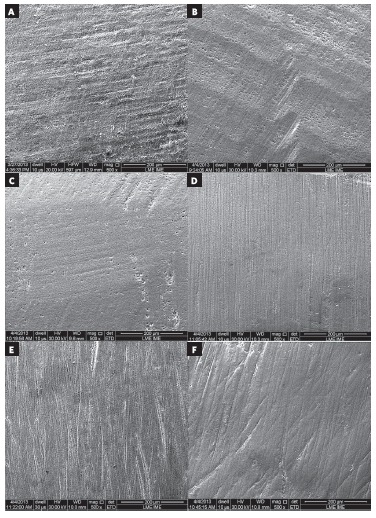
- Scanning electron microscopy (500 X magnification) showing the effect of enamel clean-up procedures on the surface. A) 12-blade tungsten carbide bur (low speed) (G12L); B) 12-blade tungsten carbide bur (high speed) (G12H); C) 30-blade tungsten carbide bur (low speed) (G30L); D) DU10CA ORTHO polisher; E) Renew Finishing System; F) Diagloss polisher.

Inspection in scanning electron microscopy shows the enamel surface before bonding (Fig 3) as well as after debonding and enamel clean-up (Fig 4). Scratches produced by the 12-blade burs at low speed are presented in Figure 4A. Deeper scratches were produced by the burs at high speed (Fig 4B). The highest degree of surface smoothness was obtained in Group G30L (Fig 4C) This group presented surface more similar to the original tooth, as shown in Figure 3. In Groups GDU and GR, there was loss of perikymata with fine scratches caused by polishers of varying abrasiveness (Fig 4D and Fig 4E). Fine scratches, which appeared to be well-marked and deep, caused by the diamond particles embedded in rubber, were also seen in Group GD (Fig 4F).

## DISCUSSION

In this study, six protocols for removal of adhesive remnant from enamel after bracket debonding were assessed. The choice of burs and abrasive points was based on the protocols most used by orthodontists, in other words, tungsten carbide burs in low and high-speed handpieces,^3^^-^ 6 and products launched on the market in recent years.

Many studies use the parameter Ra as the only indicator of surface smoothness. However, this universally accepted parameter has limitations when used alone^5^^,^ 7 because it does not determine the profile of irregularities and makes no distinction between peaks and valleys. The association of other parameters used in this study, such as Rz, enabled us to study the shape of the vertical profile.

In this study, the protocols involving 12-blade tungsten carbide burs at low and high speed produced similar results considering Ra. The Rz parameter, however, was markedly affected when the 12-blade tungsten carbide bur was used at high speed. A ΔRz value of 0.49 was the only positive value (indicating increase in roughness) and statistically different from Groups G30L and GD. This outcome demonstrated the increase in irregularities with sporadic deep scratches, which were not detected by ΔRa, because Ra is an indicator of mean roughness and does not account for the presence of an occasional peak or valley. In SEM evaluation, the 12-blade bur produced deeper scratches at high speed. However, no statistically significant difference was observed for both roughness parameters (ΔRa and ΔRz) between G12L and G12H.

The literature reports that the use of tungsten carbide burs at high speed to remove resin remnant after debonding leaves the surface similar to that of intact enamel;^2^^,^ 8 ^,^ 13 ^,^ 14 however, at the cost of a substantial loss in enamel thickness (19.2 µm).^2^^,^ 15 Other studies recommend the use of tungsten carbide burs at low speed^3^^,^ 16 ^-^ 18, which create fine scratches^19^ with a lower level of enamel loss (7.9 µm to 11.3 µm)^2^^,^ 10.

In this study, enamel loss was not measured, although this factor should be an important consideration when choosing the method for resin removal. According to Smith et al,^21^ the average enamel thickness of a maxillary central incisor is approximately 0.6 mm (600 µm). Considering a single bracket/resin removal, a loss of 10 or 20 µm might seem harmless, but it is necessary to consider the possibility of multiple rebondings due to bracket loss (caused by the patient) or bonding errors (caused by the orthodontist). Therefore, the orthodontist should minimize enamel damage and loss.

The use of high-speed burs without water cooling has been previously described by Bicakci et al.^22^ They observed heating in the pulp chamber, leading to vascular hyperemia and occasional breakage of odontoblasts. However, this condition is transient, thereby indicating that the damage caused by this protocol is reversible, and pulp repair occurs within about 20 days. The authors recommend removing most of the resin under water cooling and turning the water cooling off when removing the last layer of resin, so that it is possible to successfully distinguish between enamel and resin, thereby preventing further damage to enamel. Considering the results of the present study, low-speed burs without water cooling could be used to remove the last layer of resin, so the risk of enamel scratches might be reduced. In this study, all resin remnant was removed without water cooling. It is suggested that future studies assess enamel roughness and loss when following the aforementioned recommendations. 

Group GR, which involved the use of 12-blade tungsten carbide burs at high speed followed by Renew polisher, showed a significant (*p* < 0.05) decrease in the two roughness parameters between the two time points, indicating the importance of gently eliminating the last layer of resin with polishers after using burs at high speed.^1^^,^ 6 ^,^ 8 The literature shows that the sequential use of multiple tools for polishing is more efficient than one-step procedures^2^^,^ 3 ^,^ 17 ^,^ 20 ^,^ 23 ^,^ 24 in terms of reduction in surface roughness. In this study, GR resulted in a low level of surface roughness, with negative values for ΔRa and ΔRz. However, the final variation in ΔRz roughness of GR was not statistically different from the majority of the other groups, except for G12L (Table 4).

Roughness values obtained after clean-up in Groups G30L, GDU, GR and GD were lower than the initial roughness values. Similar results were found in other studies,^7^^,^ 18 ^,^ 20 in which abrasive points and 30-blade tungsten carbide burs were used to eliminate adhesive remnant. In a previous study, microscopic evaluation showed that the use of abrasive points (Optimize Discs - TDV - and Onegloss Discs - Shofu) maintained the enamel surface of the study groups in a similar condition to the enamel surface of the control groups.^25^

The time required for removing resin differed among the six groups, mainly due to differences in the cutting power of tools used,^7^ which was mainly determined by the speed of rotation,^17^ type of bur, number of blades, composition, particle size, and pressure applied to the handpiece.^2^ The latter variable was minimized because the same operator performed all resin removal procedures. The time required in the groups in which Diagloss polishers (63.5 seconds) and 30-blade tungsten carbide burs at low speed (57.5 seconds) were used, was significantly longer than that required in the other groups (*p* < 0.05). The protocol used in GD involved two steps: use of rubber with a high gradient of diamond particle concentration, which ensured resin reduction, followed by another point for polishing. Hence, the procedure consumed more chair time. In Group G30L, the higher number of blades of the bur used at low speed decreased its cutting power and removed the resin layer by layer, which results in a smoother and scratch-free surface; however, it increased the time required for resin removal.

The fastest protocol was the use of the 12-blade tungsten carbide bur at high speed (23.5 seconds), followed by DU10CA ORTHO polisher (31.8 seconds), and the Renew system (31.9 seconds), which also made ​​use of 12-blade tungsten carbide burs at high speed, however, in a two-step procedure. Ryf et al^20^ assessed the Renew system, and showed it required a considerably longer time to remove and polish the enamel (83.6 seconds); however, the burs were used were at low speed. The potential reasons for this difference were the use of lower-speed handpieces (under 20,000 rpm) and the use of the same bur every 10 specimens; thus, the bur became worn and had its cutting power diminished.

Our findings corroborate those of other studies,^3^^,^ 14 ^,^ 19 ^,^ 20 ^,^ 25 ^,^ 26 indicating that all rotary instruments cause varying changes in enamel surface. The association between the time spent and change in roughness (ΔRa, ΔRz) showed a negative and moderate correlation: the longer the time spent on removing the remaining resin, the lower was the roughness left on the enamel surface, which is in agreement with a previous study.^1^ Instruments with low cutting power perform slower resin removal, leaving a smoother surface less prone to plaque adhesion and pigmentation.

After orthodontic treatment, it is impossible to restore the surface of teeth to their original condition. Prophylaxis with pumice, acid etching, debonding and aggressive resin removal procedures cause enamel loss.^15^ Rotating instruments create some degree of enamel irregularities, and when rebonding is frequently necessary, the surface is modified and the perikymata pattern of young teeth is probably damaged.^3^ Therefore, fine scratches, such as those made when using the protocols tested in this study, appear to cause minimum damage and must be placed in an expected clinical perspective. It is up to the orthodontist to apply methods to minimize damage to tooth enamel.^25^ Thorough resin removal and polishing after debonding is entirely dependent on the operator^27^ who is responsible for selecting the instruments, using points with particles with a lower degree of hardness than enamel to minimize iatrogenic abrasions and scratches;^2^ for the pressure applied to the handpiece and for eliminating resin from the tooth surface.

## CONCLUSIONS

1) All finishing and polishing protocols were considered satisfactory for residual resin removal without increasing enamel roughness.
2) The time spent on enamel clean-up varied from 23.5 (12-blade tungsten carbide bur at high speed) to 63.5 seconds (Diagloss polishers).
3) The longer the time spent on removing the remaining resin, the smaller the variation in roughness level.
